# Comparative resistomics analysis of multidrug‐resistant *Chryseobacteria*

**DOI:** 10.1111/1758-2229.13288

**Published:** 2024-06-23

**Authors:** Dung Ngoc Pham, Mengyan Li

**Affiliations:** ^1^ Department of Chemistry and Environmental Science New Jersey Institute of Technology Newark New Jersey USA

## Abstract

*Chryseobacteria* consists of important human pathogens that can cause a myriad of nosocomial infections. We isolated four multidrug‐resistant *Chryseobacterium* bacteria from activated sludge collected at domestic wastewater treatment facilities in the New York Metropolitan area. Their genomes were sequenced with Nanopore technology and used for a comprehensive resistomics comparison with 211 *Chryseobacterium* genomes available in the public databases. A majority of *Chryseobacteria* harbor 3 or more antibiotic resistance genes (ARGs) with the potential to confer resistance to at least two types of commonly prescribed antimicrobials. The most abundant ARGs, including β‐lactam class A (*blaCGA‐1* and *blaCIA*) and class B (*blaCGB‐1* and *blaIND*) and aminoglycoside (*ranA* and *ranB*), are considered potentially intrinsic in *Chryseobacteria*. Notably, we reported a new resistance cluster consisting of a chloramphenicol acetyltransferase gene *catB11*, a tetracycline resistance gene *tetX*, and two mobile genetic elements (MGEs), *IS91* family transposase and *XerD* recombinase. Both *catB11* and *tetX* are statistically enriched in clinical isolates as compared to those with environmental origins. In addition, two other ARGs encoding aminoglycoside adenylyltransferase (*aadS*) and the small multidrug resistance pump (*abeS*), respectively, are found co‐located with MGEs encoding recombinases (e.g., *RecA* and *XerD*) or transposases, suggesting their high transmissibility among *Chryseobacteria* and across the *Bacteroidota* phylum, particularly those with high pathogenicity. High resistance to different classes of β‐lactam, as well as other commonly used antimicrobials (i.e., kanamycin, gentamicin, and chloramphenicol), was confirmed and assessed using our isolates to determine their minimum inhibitory concentrations. Collectively, though the majority of ARGs in *Chryseobacteria* are intrinsic, the discovery of a new resistance cluster and the co‐existence of several ARGs and MGEs corroborate interspecies and intergenera transfer, which may accelerate their dissemination in clinical environments and complicate efforts to combat bacterial infections.

## Graphical abstract

*Chryseobacteria* are resistant to β‐lactam, aminoglycoside, and many other antimicrobials. Comparative resistomics analysis based on 215 *Chryseobacteria* genomes revealed that >78% of antibiotic resistance genes are putatively intrinsic. However, the discovery of the resistance cluster *tetX‐catB11‐IS91‐XerD* and its enrichment in clinic isolates suggest transmissibility within and beyond the genus of *Chryseobacterium.*

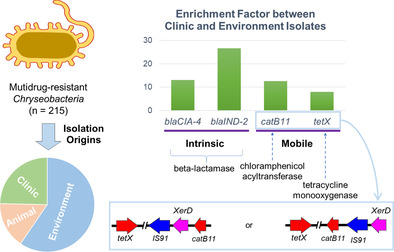

## INTRODUCTION

We are facing an unprecedented threat of antimicrobial resistance (AMR) to our health. It was estimated that 1.27 million deaths in 2019 were directly due to AMR‐related infections across the world (Murray et al., 2022). If the situation is left unsolved, AMR can cause as many as 10 million annual deaths, making it the top 1 killer by 2050 (O'Neill, 2016). Even worse, pathogenic bacteria can evolve from environmental species, particularly those that intrinsically confer resistance to clinical antimicrobials (Lopeman et al., 2019). They can also acquire new resistance via horizontal gene transfer (HGT), particularly under stress in clinical and other environments with high and frequent antimicrobial exposure (Lerminiaux & Cameron, 2019).

Members of the bacterial genus *Chryseobacterium* are among such human pathogens that are causal for a myriad of nosocomial infections, including pneumonia, bacteremia, biliary tract, and intra‐abdominal infections (Kirby et al., 2004). Increasing infection cases caused by *Chryseobacteria* have not only been observed in critically ill patients in intensive care units but also in people infected by community‐acquired *Chryseobacteria*. Particularly, infection caused by *Chryseobacterium indologenes* has remained a critical concern in Taiwan since 1992 (Hsueh et al., 1997). During 1992–1995, an epidemicity of *C. indologenes* led to 14% death in people who were infected by this pathogen (Hsueh et al., 1997). Other troublesome *Chryseobacterium* species include *Chryseobacterium gleum*, *Chryseobacterium hominis*, *Chryseobacterium arthrosphaerae,* and *Chryseobacterium oranimense*. The consistent increase of these pathogenic species in the genus of *Chryseobacterium* is evident in Africa, Asia, Europe, and America (Kirby et al., 2004; Lin et al., 2019).

Despite being clinic‐relevant, *Chryseobacteria* are also widespread in the environment and can be found in soil, plants, freshwater, municipal wastewater, and drinking water, and a number of these species are known as opportunistic human pathogens (Vandamme et al., 1994). Many environmental and clinical *Chryseobacterium* species were reported with resistance to antimicrobials and disinfectants, biofilm‐forming capacity, and metabolic versatility, promoting them to survive in diverse environments and colonize and infect humans (Dijkshoorn et al., 2007). Some *Chryseobacteria* are reported to confer resistance to commonly used antimicrobials, particularly those for treating Gram‐negative bacteria, such as carbapenems, colistin, tigecycline, and other “last‐resort” antimicrobials (Kirby et al., 2004; Mwanza et al., 2022; Zhang et al., 2020). For instance, *C. indologenes* is resistant to a broad spectrum of β‐lactams, including amoxicillin, cephalosporins, and carbapenems due to the expression of Class A (e.g., *blaCIA*) and/or Class B (e.g., *blaIND*) β‐lactamases (Chen et al., 2013). Furthermore, resistance to environmental stresses (e.g., disinfectants and heavy metals) was also reported in some *Chryseobacterium* species detected in chlorinated hospital water, downstream of France and Belgium's wastewater treatment facilities, and uranium‐contaminated soil in Northeast India (Arouna et al., 2017; Garcia‐Armisen et al., 2011; Khare et al., 2020). Even worse, a recent study isolated 13 different *Chryseobacterium* strains from food, soil, plant rhizosphere and phyllosphere, and surface water, and these isolates are putative human pathogens considering their ability to express virulence enzymes, such as α‐ or β‐haemolyses, proteases, lipases, and DNases (Mwanza et al., 2022). These characteristics of multidrug‐resistant *Chryseobacteria* revealed the potential as a significant and growing threat to global health (Dijkshoorn et al., 2007).

Response to the challenge of multidrug‐resistant *Chryseobacteria* requires a comprehensive understanding of AMR mechanisms and evolutionary traits, which will be beneficial to stimulate the development of appropriate therapeutic approaches. Some recent studies have highlighted the genotypes and phenotypes related to resistance and virulence of *Chryseobacterium* isolates (Kirby et al., 2004; Mwanza et al., 2022; Victor et al., 2022; Zhang et al., 2020). For example, Mwanza et al. investigated the pathogenicity potential of 37 *Chryseobacterium* strains isolated from clinical, fish, food, and environmental sources (Mwanza et al., 2022). Another study has centred on virulence factors and secondary metabolites of 73 *Chryseobacterium* genomes without considering their isolation sources (Victor et al., 2022). However, our current knowledge regarding resistomes and genetic factors that may promote the dissemination of AMR in *Chryseobacteria* between environmental and clinical settings remains fragmentary.

In this study, association among key antimicrobial resistance genes (ARGs), virulence factors, and other functional genes were untangled in a systematic fashion, engaging the comprehensive comparison of 215 *Chryseobacterium* genomes from three source categories (i.e., environmental, animal, and clinical settings) available in public databases, as well as those isolated in our lab from activated sludge samples collected at three municipal wastewater treatment plants (WWTPs) in the New York metropolitan area. AMR in these isolates were further validated by assessing their minimum inhibitory concentrations (MICs) to major antimicrobials that are common for clinical use. Our central hypothesis is that the multidrug resistance of *Chryseobacteria* is dominantly intrinsic, promoting its viability in diverse environments. They can also acquire and disseminate AMR via HGT with transmission potential to human pathogens of close phylogenies, such as *Elizabethkingia meningoseptica* and *Myroides odoratimimus*, underscoring their impacts in environmental and human resistomes.

## METHODS

### *Isolation of multidrug‐resistant* Chryseobacteria

Activated sludge samples were collected from aeration tanks of three WWTPs in the New York metropolitan area (i.e., L, P, and R sites) between June and October 2019. These WWTPs were selected as they are representative of WWTPs that are of different sizes and wastewater input. These WWTPs serve residential populations in the range from 6.0 × 10^4^ to 1.4 × 10^6^, as well as a diversity of domestic industries. Ten‐time dilution of activated sludge samples was spread onto R2A agar plates amended with 100 mg/L ampicillin, 50 mg/L kanamycin, 20 mg/L tetracycline, and 50 mg/L sulfamethoxazole. After incubation for 2 days at 37°C, multidrug‐resistant colonies were formed on plates. Even though seeded with activated sludge from different WWTPs, a majority of these colonies appeared yellow in colour. Based on the 16S rRNA sequencing, four of these yellow colonies were identified as members of the genus *Chryseobacterium*. This was in agreement with the ability of *Chryseobacteria* to produce flexirubin‐type pigments, which impart yellow‐orange color colonies. The resistance to these four antibiotics aforementioned for isolation was further validated for these four *Chryseobacteria* in batch cultures in R2A broth dosed at the same antibiotic concentrations. The genomic DNA of these isolates were extracted for taxonomy identification and whole‐genome sequencing. They were also tested to determine their MICs to different antibiotics and susceptibility to UV and chlorine disinfection, as detailed below.

### Nanopore sequencing and de novo assembly

High molecular‐weight DNA of four sludge‐derived *Chryseobacterium* isolates (P3, R7, L8 and N14) was extracted using the Quick‐DNA HMW Magbead kit (Zymo Research, USA), followed by the purification using AMPure XP beads (Beckman Coulter). DNA concentration and quality were examined using the SpectraMax Plus 384 Microplate Reader equipped with a SpectraDrop Micro‐volume Microplate (Molecular Device, CA). DNA samples with OD 260/280 of >1.8 and OD 260/230 of >1.9 were selected for library preparation. Whole‐genome sequencing of these isolates was prepared with the SQK‐LSK109 1D ligation genomic DNA kit (Oxford Nanopore, UK) following the instruction manual. Long‐read sequencing was performed using an Oxford Nanopore MinION flow cell (R9.4.1). Sequencing data acquisition was processed using the MinKNOW software without live base calling.

Raw reads generated from the MinION sequencing were base‐called using Guppy, and the passed reads subsequently underwent adapter and barcode trimming using Filtlong v.0.2.0. Quality of the whole‐genome sequencing was accessed using NanoPlot v.1.30.1. High‐quality reads (base call accuracy >92%) were used for de novo assembly using Flye v.2.8.3 (default settings, except—plasmids). The assembly contigs were subjected to one round of polishing by Racon v.1.4.10 (default setting, except m 8‐x‐6‐g‐8‐w 500) followed by three rounds of polishing by Medaka v.1.2.3 (Sereika et al., 2022). The quality of the genome before and after polishing was analysed using CheckM (Parks et al., 2015) and shown in Table S1. Polished genomes of the *C. indologenes* P3, *C. indologenes* R7, *C. bernardetii* L8, and *C. gleum* N14 were deposited to Joint Genome Institute (JGI; https://genome.jgi.doe.gov/portal/) under IMG genome IDs of 2886347146, 2886351778, 2886357245 and 2886362528, respectively.

### *Collection of* Chryseobacterium *genomes and quality control*

We identified 254 *Chryseobacterium*'s genomes available in the databases of the National Center for Biotechnology Information (NCBI) Sequence Read Archive (SRA) (https://www.ncbi.nlm.nih.gov/sra) and JGI that includes four genomes of our isolates aforementioned. Information on genome ID, taxonomy, and isolation source is summarized in Data S1. Quality control of these retrieved bacterial genomes was further processed to ensure high quality and minimal bias by following three procedures. First, only isolates with known isolation sources were selected for further investigation. Second, only genomes with at least 95% completeness and less than 5% contamination were used after the examination by CheckM v1.2.1 (Parks et al., 2015). Third, to remove the redundant genomes, FastANI v1.3.2 (Jain et al., 2018) was employed to calculate the whole‐genome average nucleotide identity (ANI) and percentage of orthologous matches. The genomes were considered duplicates when the ANI value was >99.995% and the matching percentage was >90% (Levy et al., 2018). Through these three procedures, 215 high‐quality and non‐redundant genomes of *Chryseobacterium* isolates were obtained, including 128, 34, and 53 strains isolated from environment, animal, and clinical settings, respectively.

### Bioinformatics analysis

For ARG annotation, two approaches were used: (1) the amino acid sequences from *Chryseobacterium* genomes were searched against the Comprehensive Antibiotic Resistance Database (CARD) (Alcock et al., 2020) and ARG‐ANNOT (Gupta et al., 2014) using Blastp with identity and query coverage thresholds of 50% and 50%, respectively; (2) AMRFinder v.3.8.4 (Feldgarden et al., 2019) was used to screen ARGs in the isolate genomes with the same thresholds. Redundant ARGs were manually eliminated from the combined data set. Orthologs of protein‐coding sequences were screened using Proteinortho v.6.0.23 with default parameters (Lechner et al., 2011), among which genes that appeared in all 215 bacterial isolates were identified as the core genes of the genus *Chryseobacterium* as listed in Data S2 (Levy et al., 2018). KEGG Orthology IMG database of core genes was subsequently used to construct KEGG pathways using the KEGG mapper (https://www.genome.jp/kegg/mapper/).

G + C contents and mobile genetic elements (MGEs) within 10‐kpb flanking regions of ARGs were determined to identify if certain ARGs are intrinsic. Theoretically, intrinsic ARGs have (1) similar G + C contents to those of core genes detected in their hosts, (2) are chromosomally encoded and (3) are non‐mobilized genes. An ARG is considered mobile if one or more MGEs are located within 10 kbp of its flanking region or it is located on a plasmid. The G + C contents of ARGs and core genes were calculated using the bioawk toolkit. MGEs were determined by a string that matches one of the following keywords in the description of genes: integron, integrase, relaxase, type IV coupling, type IV secretory, replication initiator, transposase, transposon, conjugation, conjugative, recombinase, conjugal, mobilization, recombination, and excisionase (Li et al., 2017). The GFF files of IMG and NCBI isolates were converted to BED files, which were further used to search for 10‐kbp‐flanking regions of ARGs by the Bedtools window (v.2.26.0). In addition, to investigate if ARG located on a plasmid, ARG‐containing contigs were searched (1) against the NCBI plasmid database PLSDB (version 2020_06_23) with a minimum similarity of ≥95% and a query coverage of ≥60%, and (2) to analyze if plasmid related genes are present using a keyword of ‘plasmid’ in the description of genes.

Phylogenetic trees of 215 *Chryseobacterium* genomes were constructed based on ARGs and virulent factor profiles using PhyloPhlAn v.3.0.51 (Segata et al., 2013). Phylogeny and the distribution of ARGs were visualized with an online tool iTOL v6 (http://iTOL.embl.de/) (Letunic & Bork, 2021).

### Statistical analysis

The distribution of ARGs, virulent factors, and ortholog genes across the origins (i.e., the environment, animals, and clinical setting) was statistically compared using Fisher's exact test based on the presence or absence of a specific gene in bacterial genomes (Xia et al., 2018). FDR‐corrected *p* values according to Benjamini–Hochberg (a false discovery rate multiple test correction) (Benjamini & Hochberg, 1995) were subsequently estimated. Significant enrichment of certain genes in origin was defined when the adjusted *p* value was >0.001 compared to another origin. Fisher's exact test was performed using the MASS package within R v4.1.2 (R Core Team, 2013).

Correlation of *Chryseobacteria* isolated from different origins based on their resistome was performed using Bray Curtis distance within QIIME2 (Bolyen et al., 2019; Bray & Curtis, 1957). Pairwise permutational multivariate analysis of variance (pairwise perMANOVA) was applied to evaluate the significance of differences in *Chryseobacterium* isolates between each pair of origins using the adonis function (Anderson, 2001). FDR‐corrected *p* values were estimated for pairwise perMANOVA tests.

### Antimicrobial susceptibility test

MIC test of our four isolates against ampicillin, cefotaxime, imipenem, kanamycin, gentamycin, chloramphenicol, tetracycline, and rifamycin were determined using broth micro‐dilution technique according to CLSI recommendations (Patel et al., 2015). *Escherichia coli* ATCC 25922 and *Pseudomonas aeruginosa* ATCC 27853 were used as reference strains for the quality control for the MIC test.

## RESULTS

### *A majority of ARGs detected in 215* Chryseobacterium *isolates are potentially intrinsic*

**FIGURE 1:**
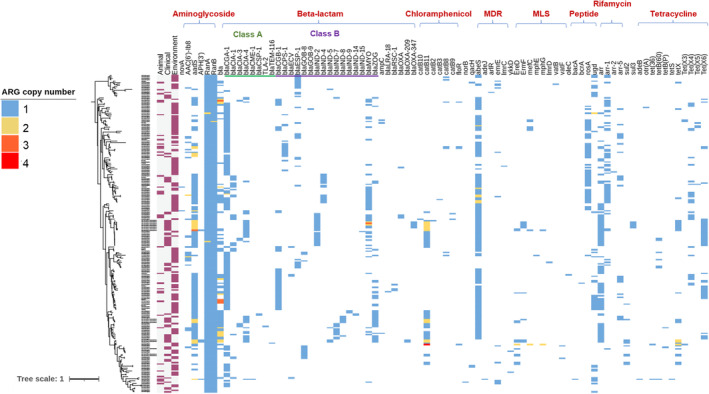
A phylogenetic tree of 215 *Chryseobacterium* genomes based on the resistomes. The left column depicts the three sources of *Chryseobacterium* isolates (i.e., animal, clinical and environment), and the right heat map shows the abundance of antibiotic resistance genes (ARGs) found in the genomes of these isolates.

*Chryseobacteria* were identified to carry a wide spectrum of ARGs based on the investigation of 215 *Chryseobacterium* genomes available in the databases of JGI and NCBI. As shown in Figure 1, a total of 82 ARG subtypes belonging to 13 ARG types were detected. Notably, almost all isolates (~98%) contained 3 or more ARGs and confer resistance to at least two different types of antimicrobials. Particularly, an environmental isolate *Chryseobacterium* sp. POL2 (IMG genome ID of 2,888,279,485) harbors as many as 19 ARGs in its genome.

Among 82 ARG subtypes, genes conferring resistance to aminoglycosides (*aadS*, *RanA*, *RanB*), β‐lactams (*blaCIA* variants, *blaCGA*, *blaCGB*, *blaIND* variants, *blaMYO*), chloramphenicol (*catB* variants), peptide antibiotics (*rosA*), rifamycins (*arr* variants), tetracyclines (*tetX* variants), and multi‐drugs (*abeS*) were found in >20% of 215 *Chryseobacterium* genomes (Table 1). Given their prevalence in *Chryseobacteria*, these ARGs were considered key ARGs and selected for further analysis. The antibiotic resistance mechanisms of these ARGs were annotated as either antibiotic inactivation or efflux pump.

**TABLE 1:** Characteristics of key ARGs of *Chryseobacteria* about their detection frequency, G + C content, percentage of mobile ARGs, and major MGEs detected within 10 kbp of individual ARGs.

Antibiotic	ARG	Mechanism	Number of ARGs	G + C content (median, %)	Mobile ARGs (%)	Adjacent MGEs	Mobility	Extrinsic possibility
Aminoglycosides	*aadS*	Inactivation	134	37.0	**36**	Recombinase *RecA*/*XerD* and transposase	High	High
	*RanA*	Efflux pump	217	35.7	3	Transposase	Low	Low
	*RanB*	Efflux pump	216	37.7	3	Transposase	Low	Low
β‐Lactams	*blaCGA‐1*	Inactivation	122	35.9	3	Transposase	Low	Low
	*blaCIA* variants	Inactivation	59	36.9	0		Low	Low
	*blaCGB‐1*	Inactivation	43	38.2	14	Recombinase *XerD*	Medium	Medium
	*blaIND variants*	Inactivation	57	39.1	0		Low	Low
	*blaMYO*	Inactivation	54	37.3	4	Transposase and recombinase *XerD*	Low	Low
Chloramphenicol	*catB* variants	Inactivation	70	37.8	**57**	Recombinase *XerD/XerC*	Very high	Very high
Multidrug	*abeS*	Efflux pump	145	38.6	15	Transposase	Medium	Medium
Peptide antibiotics	*rosA*	Efflux pump	51	37.5	4	Transposase	Low	Low
Rifamycins	*arr* variants	Inactivation	148	**40.1**	11	Recombinase and integrase	Medium	Very high
Tetracyclines	*tetX*	Inactivation	26	37.4	**46**	*IS91* family transposase	Very high	High
	*tet(X3)/(X4)/(X6)*	Inactivation	72	**40.8**	3	Transposase	Low	Very high

Approximately 78% of total key ARGs, which include *ranA, ranB*, *blaCIA* variants, *blaCGA*, *blaCGB*, *blaIND* variants, *blaMYO*, *abes*, *rosA,* and *arr* variants, were potentially intrinsic in *Chryseobacteria*, since (1) none of ARGs are located on plasmids as no ARGs carried contigs were identified as plasmids based on the database analysis, (2) their G + C contents were in a similar range to those of core genes that were shared by all 215 *Chryseobacteria* genomes (35.9%–40.1% vs. 37.6%) and (3) less than 20% were putatively mobile (Table 1). It is important to note that approximately 50% of *Chryseobacterium* isolates carry both Class A (i.e., *blaCIA* variants or *blaCGA*) and Class B (*blaCGB*, *blaIND* variants or *blaMYO*) β‐lactamase genes, accounting for 45%, 50%, and 62% of bacteria with origins from the environment, animal, and clinical settings, respectively. In addition, *ranA* and *ranB*, which encode an ABC‐type efflux pump system to exclude aminoglycoside from the bacterial cells, were detected in all 215 *Chryseobacteria* genomes. Another important intrinsic gene is *rosA*, which was demonstrated as a part of the efflux pump system (*rosAB*), enabling resistance to cationic peptide antibiotics in *Yersinia* or other bacteria (Bengoechea & Skurnik, 2000).

Phenotype analysis confirmed the multidrug resistance for all four sludge‐derived *Chryseobacterium* isolates we obtained in our lab, including *C. indologenes* P3, *C. indologenes* R7, *Chryseobacterium bernardetii* L8 and *C. gleum* N14 (Table 2). They exhibited high resistance to different classes of β‐lactam, including ampicillin (MIC ≥ 128 mg/L), cefotaxime (MIC ≥ 32 mg/L) and imipenem (MIC ≥ 8 mg/L), as well as other commonly used antimicrobials, kanamycin (MIC ≥ 16 mg/L), gentamicin (MIC ≥ 4 mg/L) and chloramphenicol (MIC ≥ 16 mg/L). All four *Chryseobacterium* isolates showed susceptibility to rifamycin, even though they contain *arr*/*arr‐5* encoding rifampin ADP‐ribosyltransferases that are known for the inactivation of this antimicrobial. This observation suggests these *arr*/*arr‐5* genes might be silent, namely cryptic, or confer other functions in our *Chryseobacterium* isolates.

**TABLE 2:** Antimicrobial susceptibility profiles of 4 sludge‐derived *Chryseobacterium* isolates.

Isolate	β‐Lactams			Aminoglycosides		Chloramphenicol	Tetracycline	Rifamycin
	Penams	Cephalosporins	Carbapenems					
	Ampicillin	Cefotaxime	Imipenem	Kanamycin	Gentamycin			
P3	128	64	64	64	8	16	16	0.5
R7	128	64	64	16	4	16	16	<0.25
L8	>256	64	64	256	32	16	32	<0.25
N14	256	32	8	64	32	32	32	2

Abbreviations: L8, *Chryseobacterium bernardetii* L8; N14, *Chryseobacterium gleum* N14; P3, *Chryseobacterium indologenes* P3; R7, *Chryseobacterium indologenes* R7.

### *Six ARGs enriched in clinical* Chryseobacterium *isolates*

**FIGURE 2:**
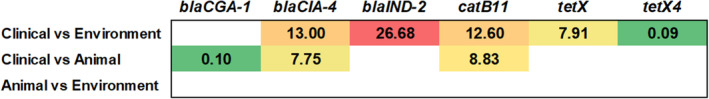
Significant differences in key ARGs between different sources. The heatmap shows the level of enrichment based on Fisher's exact test with adjusted *p* < 0.001.

Pairwise perMANOVA test revealed a significant enrichment of several *Chryseobacterium*‐carrying ARGs in clinical isolates as compared to environmental or animal isolates and (FDR‐corrected *p* = 0.003), while no significant difference was observed between environmental and animal isolates (FDR‐corrected *p* = 0.280). Approximately 6 of 82 ARG subtypes, including *blaCGA‐1*, *blaCGA‐4*, *blaIND‐2*, *catB11*, *tetX,* and *tetX4*, were significantly different across the origins (Figure 2). Particularly, *catB11* and *tetX* were found to be significantly enriched by approximately one order of magnitude in clinical isolates as compared to those with environmental origins where exposure to anthropogenic interferences is less in general (Fisher's exact test, adjusted *p* < 0.001) (Figure S1). Specifically, 54.2% of clinical isolates contain *catB11*, while only 8.3% of environmental isolates carry the same gene. Similarly, 41% of clinical isolates contain *tetX*, while only 4.5% of environmental isolates carry this ARG. Enrichment of *catB11* at a factor of 8.83 was also evident in clinical isolates in comparison to animal isolates. 54.2% of clinical isolates contain *catB11*, whereas it is only found in 10.5% of animal isolates.

### *Three ARGs with transmission potentials within the* Bacteroidota *phylum*

Though the majority of ARGs are putatively intrinsic, three ARGs (Table 2) harbored by *Chryseobacteria* were identified with high potential to be transmissible among species within the *Bacteroidota* phylum, including *catB11*, *tetX*, and *aadS*, which confer resistance to chloramphenicol, tetracycline, and aminoglycosides, respectively.

**FIGURE 3:**
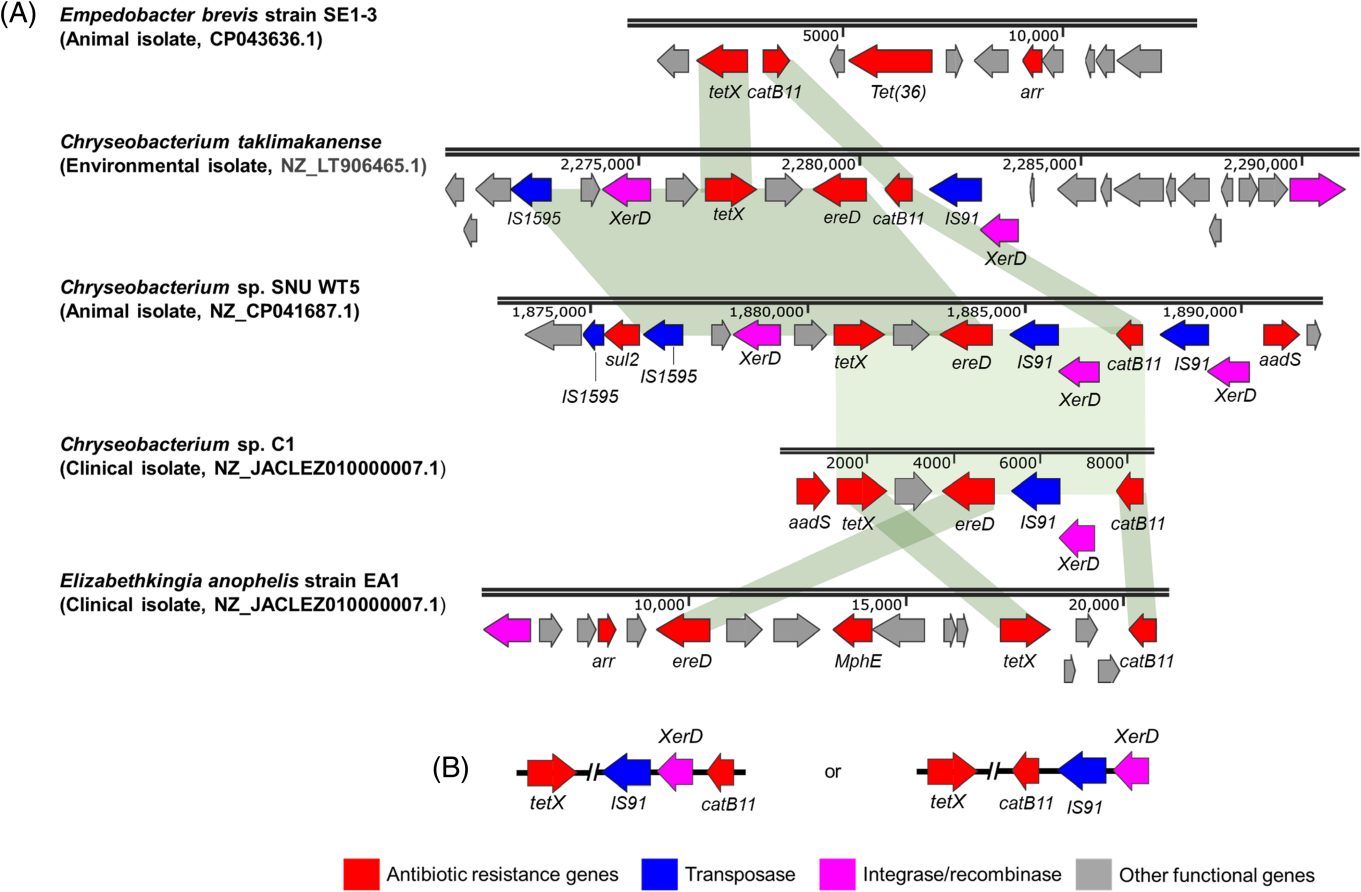
(A) Comparative analysis between the *catB‐tetX*‐flanking regions in *Chryseobacterium* genomes and those from the genomes of other species in the *Bacteroidota* phylum. Shades show conserved regions of higher than 94% similarity in nucleotide sequences. (B) Two models of *tetX‐catB11* resistance clusters.

Genome mining of *catB11* and *tetX* in 215 *Chryseobacterium* genomes and other bacterial genera from the NCBI non‐redundant (nr) database revealed a resistance cluster consisting of both *catB11* and *tetX* genes in 9 *Chryseobacterium* isolates from different sources with high similarity of >90% (Table S2). As shown in Figure 3, this resistance cluster is often located next to the *IS91* family transposase and tyrosine‐type recombinase *XerD* in the *Chryseobacterium* genomes. The co‐occurrence of *catB11* and *tetX* was also identified in other species, such as *Empedobacter brevis* strain SE1‐3 and *Elizabethkingia anophelis* strain EA1 (Figure 3). However, this co‐existence is not common in other *Bacteroidota*, even though the *tetX* gene (WP_008651082.1) is frequently detected in 15 genera belonging to the *Bacteroidota* phylum, as well as the *Aliarcobacter* genus of the *Proteobacteria* phylum. Meanwhile, *catB11* (WP_035589996.1) is less frequent and detected in <5 genera of the *Bacteroidota* phylum.

**FIGURE 4:**
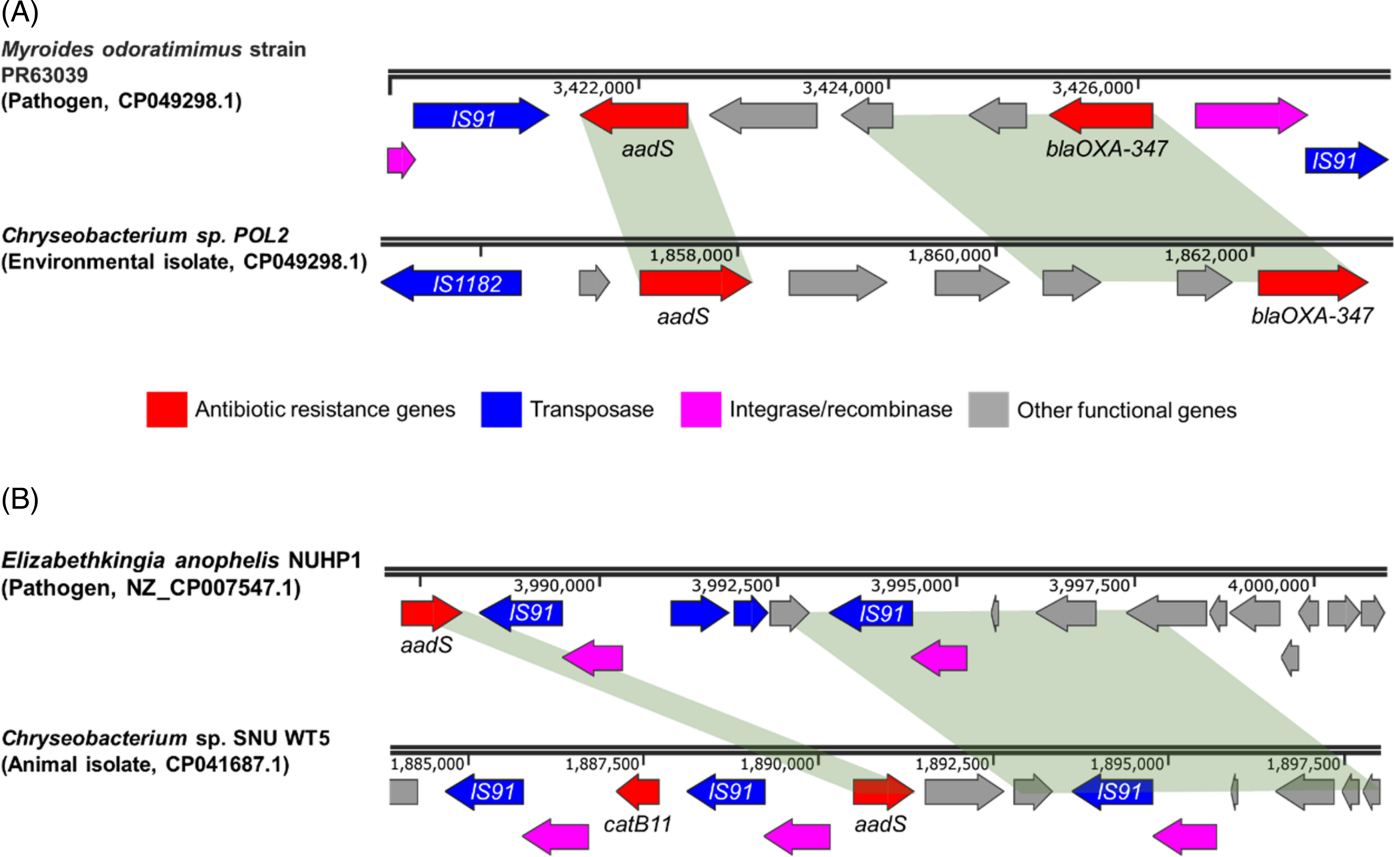
Comparative analysis between the *aadS*‐flanking regions in *Chryseobacterium* genomes and those from the genomes of other species in the *Bacteroidota* phylum. Shades show conserved regions of higher than 98% similarity in nucleotide sequences.

Approximately 35% of 134 *aadS* genes co‐existed with MGEs encoding recombinases (e.g., *RecA* and *XerD*) or transposases. As depicted in Figure 4A, the environmental isolate *Chryseobacterium* sp. POL2 carries an *aadS* gene that is 100.0% identical to that identified in the pathogen *M. odoratimimus* strain PR63039. The *aadS* gene in POL2 is also located in a 2.7‐kbp flanking region that overlaps with the genome of PR63039, implying these sequences between these two bacteria may share an identical origin. Similarly, the genome of an animal isolates *Chryseobacterium* sp. SNU WT5 shared >99.4% identity to both *aadS* and the 8.6‐kbp region in the pathogen *E. anophelis* NUHP1 (Figure 4B). *AadS* genes (NCBI accession number of AAA27459.1) were not only identified in *Chryseobacteria*, but also in 13 other genera that belong to the *Bacteroidota* phylum according to NCBI identical protein groups. High similarity (>98%) of *aadS* genes and their flanking regions between *Chryseobacteria* and other species within the *Bacteroidota* phylum (e.g., *Myroides*, *Elizabethkingia, Bacteroides* and *Riemerella*) is detailed in Table 3. These results suggest HGT of *aadS*‐containing fragments among *Bacteroidota* regardless of their pathogenicity (Forsberg et al., 2012).

**TABLE 3:** *AadS*‐carrying contigs in *Chryseobacteria* with high similarity to those detected in pathogenic species within the *Bacteroidota* phylum.

*Chryseobacterium* contig (ID: start–stop)	Closet pathogen contig/genome (NCBI accession number)	Contig		*Aads (nt)*		Pathogenic species	Host of pathogen
		Identity (%)	Length (bp)	Identity (%)	Length (bp)		
Ga0443270_01: 1880528–1901391	NZ_CP007547.1	100	8576	99.4	864	*Elizabethkingia anophelis*	Human
NZ_JACLEO010000042.1: 1–8102	NZ_CP013690.1	99.7	3046	99.8	864	*Myroides odoratimimus*	Human
Ga0439802_01: 1847255–1868118	NZ_CP013690.1	99.9	2733	99.8	864	*Myroides odoratimimus*	Human
NZ_JACLDL010000004.1: 152674–173537	NZ_RWHY01000040.1	99.3	1370	99.8	864	*Bacteroides thetaiotaomicron*	Human
NZ_JACLDY010000004.1: 245788–266651	NZ_RWHY01000040.1	99.3	1370	99.8	864	*Bacteroides thetaiotaomicron*	Human
Ga0347461_03: 923406–939449	NZ_CP072188.1	99.4	1060	99.8	864	*Riemerella anatipestifer*	Goose
LX71DRAFT_scaffold00046.46: 11368–24032	NZ_QXFQ01000008.1	100	1037	99.8	864	*Riemerella anatipestifer*	Duck
Ga0127023_107: 1–11064	NZ_QXFQ01000008.1	100	1037	99.8	864	*Riemerella anatipestifer*	Duck
NZ_JACLCU010000008.1: 1–19917	NZ_RWHY01000040.1	100	1054	99.8	864	*Bacteroides thetaiotaomicron*	Human

Collectively, the co‐occurrence of three ARGs mentioned above (i.e., *catB11*, *tetX,* and *aadS*) and MGEs (e.g., recombinase and transposase genes) and their high identity among *Chryseobacteria* and other members of the *Bacteroidota* phylum underline their critical role in disseminating AMR to aminoglycosides, chloramphenicol, and tetracycline.

### Mobility of a multidrug resistance gene abeS associated with a RayT transposase gene

**FIGURE 5:**
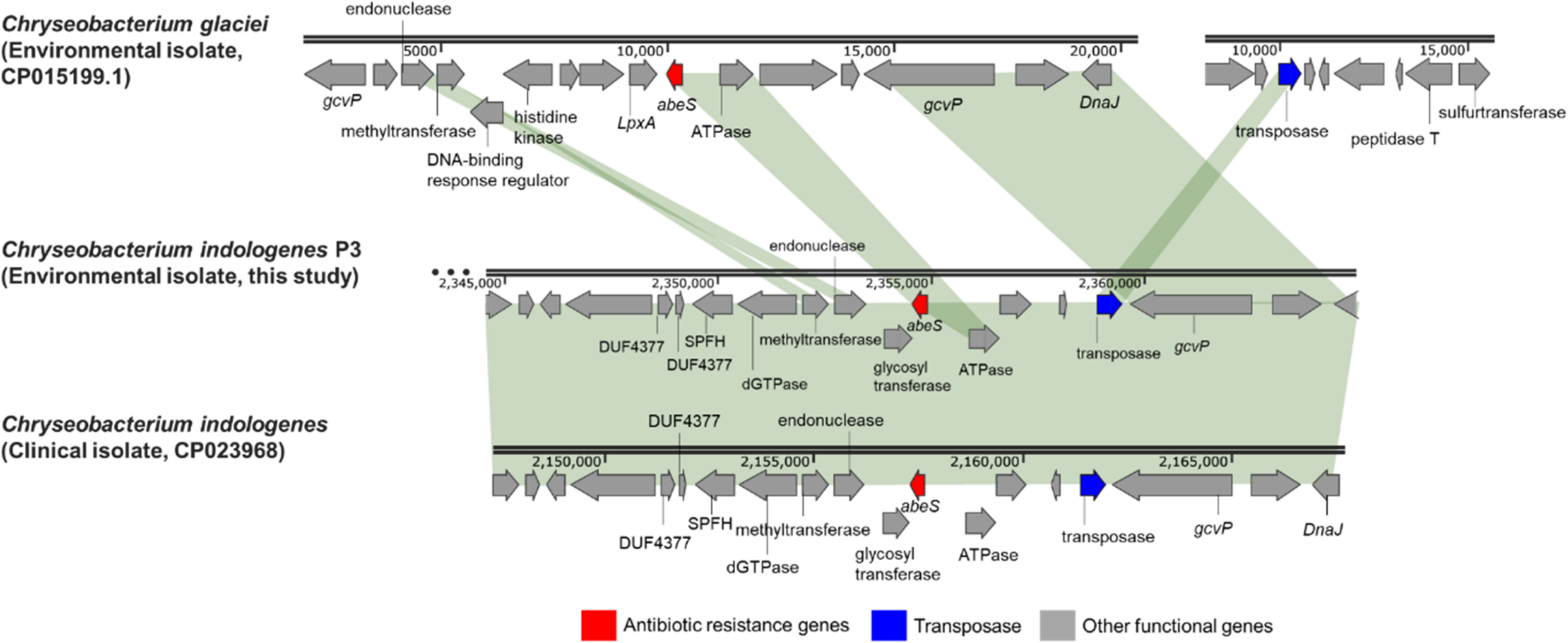
Potential genome rearrangements in the flanking regions of *abeS* based on the comparative analysis between the environmental and clinical *Chryseobacterium* isolates.

A putative insertion of the transposase gene *RayT* to 10‐kbp‐flanking regions of *abeS* was observed in many *Chryseobacteria* of clinical origins with previous anthropogenic disturbance (Figure 5). *AbeS* encodes a small multidrug resistance pump that enables bacteria to confer resistance to aminocoumarins and macrolides and this ARG was detected in 139 *Chryseobacterium* isolates (Srinivasan et al., 2009). The colocation of both *abeS* and *RayT* was found in 15 clinical and 2 environmental isolates (Table S3). Fisher's exact test revealed a significantly higher abundance of this *abeS‐RayT* cluster in clinical isolates as compared to those detected in environmental isolates (FDR *p* < 0.05). *Chryseobacteria* carrying the *abeS‐RayT* cluster were isolated from clinical settings, activated sludge, and Populus root rhizosphere, which are often related to antimicrobial exposure history (Table S3) (Chen et al., 2019; Ju et al., 2019). Meanwhile, *Chryseobacteria* without the *abeS‐RayT* cluster were often associated with environments with little anthropogenic activities, such as glaciers, soil and leaf.

As shown in Figure 5, in two *C. indologenes* isolates, the *RayT* transposase is located within 10 kbp at the flanking region of *abeS*. In contrast, this MGE is >1800 kbp away from the *abeS* gene in the chromosome of *Chryseobacterium glacier* species isolated from a glacier. Furthermore, *RayT* transposase genes located within the 10‐kbp flanking region of *abeS* exhibited much higher similarities (>74.4% for amino acid sequences) than other *RayT* transposase genes identified in *Chryseobacterium* genomes. Phylogenetic analysis revealed that these *RayT* genes can cluster into an ortholog, suggesting they might have derived from a common ancestor (Lechner et al., 2011). Genome mining also revealed the *abeS* and its cluster with the transposase gene *RayT* shared <81.0% amino acid sequence similarity with bacterial genomes other than the *Chryseobacterium* species, suggesting this *abeS‐RayT* cluster might have been recently formed within the genus *Chryseobacterium* via interpopulation transmissions (Jiang et al., 2017).

### Majority of virulence factors independent of the origins

In the 215 *Chryseobacterium* genomes, a total of 4677 genes were predicted and classified into 83 virulence factors. A majority of virulent factors were clustered into groups of adherence, immune response, mobility, nutritional/metabolic factor, and stress survival (Figure S2). Similar to the ARG profile, the majority (>93%) of predicted virulence factors were not significantly enriched in the clinical isolates as compared with other isolates from animals and environment (Table S4). Furthermore, no distinctive clusters were identified among *Chryseobacterium* isolates from environmental, animal, and clinical sources regarding the phylogenetic analysis based on virulence factor profile (Figure S2), corroborating the close relevance of environmental, animal, and clinical isolates regarding their pathogenicity.

The attachment of *Chryseobacteria* to host cells is the first critical step for infection and is associated with adherence genes, such as *Hsp60*, *EF‐Tu*, and *IlpA*. It is important to note that *Hsp60* was found in all 215 *Chryseobacterium* genomes. Following the primary surface adhesion, the growth of attached bacteria such as *Chryseobacteria* can be mediated by factors relevant to immune response, mobility (e.g., *flmH*), nutritional/metabolic traits (e.g., *mgtB*, *panD*). In *Chryseobacteria*, an immune response is predominant by the expression of capsule and formation of lipopolysaccharide (LPS) and lipooligosaccharide (LOS). Several highly abundant genes involved in the capsule biosynthesis pathway include *cap8E*, *cap8G,* and *tviB*. In addition, *acpXL*, *galE*, *rffG*, *wbpD*, *hisF2*, *lpxA*, *lpxD*, *rfbC*, *wbtL,* and *wbtF*, which are involved in the biosynthesis of LPS/LOS, were frequently detected among *Chrseobacterium* species. Several virulence factors associated with stress survival enabling bacterial pathogens to persist in host cells were also detected in *Chryseobacteria*, including *ClpP*, *UreB*, *UreG*, *KatG*, *KatAB*, *KatA*, and *icl*. *ClpP* was detected in all 215 *Chryseobacterium* isolates. Detailed discussions regarding these virulent factors are provided in the Supporting Information.

## DISCUSSION

This study was initiated by our observation that *Chryseobacteria* were dominant multidrug‐resistant bacteria screened by the plating method among different activated sludge samples collected in the New York Metropolitan area. Their resistance to commonly prescribed antimicrobials was further confirmed by MIC assays and the detection of corresponding ARGs in the genomes of these four isolates obtained in our lab. This consensus among our isolates stimulated our interest beyond these isolates to deepen our understanding of resistomes of *Chryseobacteria* at the genus level.

To date, though many emerging antimicrobial‐resistant pathogens have been identified (Sanz‐García et al., 2021; Vouga & Greub, 2016), only a few studies have performed a thorough comparison between clinical and non‐clinical species, such as *Mycobacterium abscessus* (Davidson et al., 2022), *P. aeruginosa* (Rossi et al., 2021), and *Herbaspirillum seropedicae* (Faoro et al., 2019). Considering the multidrug resistance of *Chryseobacteria* and potential pathogenicity, we conducted a comparative genomics analysis based on genomes available at NCBI and other databases in response to their origins categorized as environmental, animal, and clinical. It is notable that approximately 98% of 215 *Chryseobacterium* genomes contained more than three ARGs and the majority of them were intrinsic (Figure 1; Table 1). Intrinsic ARGs are recognized to be stably maintained in *Chryseobacteria* so they can retain the resistance with less regard to the change in the environment and growth condition (Mançano et al., 2020). Up to 50% of *Chryseobacterium* isolates carry both Class A and B β‐lactamase genes, such as penams, cephalosporins, and carbapenems, and even the last‐resort antibiotics imipenem and meropenem. This trait probably enabled *Chyseobacterium* species to confer resistance to a broad spectrum of β‐lactams. This is in good agreement with previous epidemiological analyses of *Chryseobacteria* isolated from Asian, European, and American hospitals in different investigation periods during 1994–2019 (Chen et al., 2013; Kirby et al., 2004; Zhang et al., 2020). For instance, >90% of 215 clinical *C. indologenes* strains conferred resistance to all tested β‐lactams, including the last‐resort antibiotics imipenem and meropenem (Chen et al., 2013). In addition, our discovery of the frequent existence of *rosA* in *Chryseobacterium* genomes may explain the resistance to colistin reported previously, as this ARG encodes a cationic antimicrobial peptide that is important for cystic fibrosis treatments (Sharma et al., 2015).

Phenotype analysis confirmed the resistance for all four sludge‐derived *Chryseobacterium* strains isolated in our lab to commonly used antibiotics except rifamycin despite the presence of *arr* and *arr‐5* genes (Table 2). The presence of cryptic ARGs such as *arr* and *arr‐5* genes is common in bacteria. For example, 16% of *Acinetobacter baumannii* strains contained *blaOXA‐23* but remained sensitive to imipenem (Deekshit & Srikumar, 2022). However, cryptic ARGs can be activated through various mechanisms, such as (1) mutagenesis triggered by the exposure to the corresponding antibiotics, (2) HGT to other species that harbour a positive transcriptional regulator or non‐defective promoter of acquired ARGs and (3) change of the growth medium (Deekshit & Srikumar, 2022; Stasiak et al., 2021).

Some non‐intrinsic ARGs showed a significant enrichment between clinical and environmental/animal *Chryseobacteria* species, implying the acquisition of new ARGs to clinical isolates and their potential to transmit these ARGs via HGT. In our study, a resistance cluster of *catB11‐tetX* was significantly enriched in clinical isolates as compared to those with environmental origins (Figure 2). The selection for the *catB11*‐*tetX* resistance cluster may be due to some long‐term use of antimicrobial cocktails with chloramphenicol and tetracycline for bacterial infection in the hospital. For example, six clinical‐related *Chryseobacterium* strains carrying *catB11‐tetX* clusters were isolated during the period of 2004–2019 from seven different hospitals in China. The clinical usage of chloramphenicol and tetracycline in China was estimated at approximately 215 and 1170 tons per year in 2013, respectively (Zhang et al., 2020). Interestingly, several other ARGs are also found within or in proximity to these *catB‐tetX* resistance clusters, including *ereD*, *aadS*, *aar*, *MphE,* and *sul2* (Figure 3A). For instance, *ereD* was found in four out of nine *catB‐tetX* resistance clusters in *Chryseobacteria*. The co‐existence of *catB11*, *tetX,* and other ARGs may promote a greater ecological fitness, thereby increasing the adaptability of bacteria to extensive stresses under clinical conditions.

Furthermore, *catB11* and *tetX* are located in the proximity of recombinase and transposase genes (e.g., *IS91* and *XerD*) (Figure 3B), promoting HGT of these ARGs via mechanisms, such as site‐specific recombination and random relocation, concurrently spreading both ARGs and many others (Partridge et al., 2018; Zhang et al., 2015). This resistance cluster of *catB11‐tetX*, along with recombinase and transposase genes, are also identified in other genera within the Bacteroidota phylum (Figure 3), suggesting interspecies and intergenera dissemination. Besides pathogenic *Chryseobacterium* species, many *Bacteroidota* species such as *E. meningoseptica*, *Bacteroides fragilis*, *M. odoratimimus* can cause serious infections that are often associated with high mortality rates due to their resistance to many antimicrobials (Wexler, 2007; Zhang et al., 2020). The co‐existence of ARGs and associated MGEs in *Bacteroidota* members calls for attention, considering the increased mobility and associated health impact of these ARGs. Previous studies have revealed a multitude of resistance gene clusters in different bacteria (Partridge et al., 2018). For instance, a cassette consisting of a resistance gene cluster containing disinfectant resistance gene (*qacE*) and sulfonamide protection gene (*sul1*) and the *Tn402*–*intI1* hybrid have been identified to be widespread in a wide variety of environmental and pathogenic bacteria in different habitats (Gillings et al., 2015).

The small multidrug resistance pump (*abeS*) may also be a response to previous anthropogenic interference since the insert of the *RayT* transposase gene to the flanking regions of *abeS* significantly occurred at a higher frequency in clinical isolates than those in environmental isolates (Figure 5; Table S3). Multidrug resistance pumps such as *abeS* are often considered as native elements in bacteria and not commonly detected next to the MGEs such as transposons (Alekshun & Levy, 2007). Recent studies have revealed the persistence of efflux pump systems in bacterial genomes can extend resistance to a multitude of antimicrobials, such as fluoroquinolones, β‐lactams, tetracyclines, and phenicols (Alekshun & Levy, 2007; Tikhonova et al., 2011). Therefore, the colocation of *abeS* and *RayT* in certain *Chryseobacteria* species may promote bacterial viability and proficiency when exposed to the stress of a broad spectrum of antimicrobials.

In addition to ARGs, we also found a number of pathogenicity‐associated genes potentially encoding adhesion, capsular, LPS/LOS, and virulent extracellular enzymes frequently detected among the *Chryseobacterium* isolates (Figure S2). Therefore, our study has identified multidrug‐resistant *Chryseobacteria* as potential emerging pathogens with a comprehensive understanding of their resistome and virulence. The discovery of a new resistance cluster *tetX‐catB11‐IS91‐XerD* and the colocation of *abeS* and *RayT* facilitate future investigation of resistance transfer mechanisms, ongoing transmission patterns, and genomic rearrangement events among the members that belong to or are beyond this genus, and subsequently promote the development of new antimicrobial therapy for infectious diseases caused by *Chryseobacterium* species. Furthermore, we identified prevalent ARGs and virulence factors of the *Chryseobacterium* genus, such as *ranA*, *ranB*, *Hsp60,* and *ClpP*, which can be employed to timely track the abundance and dynamics of *Chryseobacteria* in contamination events and human disease cases, leading to efficient monitoring and mitigation of future outbreaks. In addition, ARGs and virulence factors that were significantly enriched in clinical‐related *Chryseobacteria* (e.g., *catB11*, *tetX,* and *icl*) can be used as biomarkers to track the pathogenic *Chryseobacterium* populations. Beyond providing an evolutionary perspective for clinically relevant versus other *Chryseobacteria*, our study also offers a comparative resistomics strategy for investigating the evolution of other human pathogens of emerging concern.

## AUTHOR CONTRIBUTIONS

**Mengyan Li:** Supervision; resources; project administration; writing – review and editing; conceptualization; funding acquisition. **Dung Ngoc Pham:** Investigation; writing – original draft; methodology; validation; visualization; data curation; formal analysis; software.

## CONFLICT OF INTEREST STATEMENT

The authors declare no conflict of interest.

## Supporting information


**Data S1.** Supporting Information.

**Data S2.** Supporting Information.

**Data S3.** Supporting Information.

## Data Availability

The data that support the findings of this study are openly available in *Chryseobacteria* at https://genome.jgi.doe.gov/portal/, reference number 2886347146. https://img.jgi.doe.gov/cgi‐bin/m/main.cgi?section=TaxonDetail&page=taxonDetail&taxon_oid=2886347146.

## References

[emi413288-bib-0001] Alcock, B.P. , Raphenya, A.R. , Lau, T.T. , Tsang, K.K. , Bouchard, M. , Edalatmand, A. et al. (2020) CARD 2020: antibiotic resistome surveillance with the comprehensive antibiotic resistance database. Nucleic Acids Research, 48(D1), D517–D525. Available from: 10.1093/nar/gkz935 31665441 PMC7145624

[emi413288-bib-0002] Alekshun, M.N. & Levy, S.B. (2007) Molecular mechanisms of antibacterial multidrug resistance. Cell, 128(6), 1037–1050. Available from: 10.1016/j.cell.2007.03.004 17382878

[emi413288-bib-0003] Anderson, M.J. (2001) A new method for non‐parametric multivariate analysis of variance. Austral Ecology, 26(1), 32–46. Available from: 10.1063/1.2830030

[emi413288-bib-0004] Arouna, O. , Deluca, F. , Camara, M. , Fall, B. , Diallo, A.B. , Docquier, J.‐D. et al. (2017) *Chryseobacterium gleum* in a man with prostatectomy in Senegal: a case report and review of the literature. Journal of Medical Case Reports, 11, 118. Available from: 10.1186/s13256-017-1269-4 28438192 PMC5402668

[emi413288-bib-0005] Bengoechea, J.A. & Skurnik, M. (2000) Temperature‐regulated efflux pump/potassium antiporter system mediates resistance to cationic antimicrobial peptides in Yersinia. Molecular Microbiology, 37(1), 67–80. Available from: 10.3389/fmicb.2019.02083 10931306

[emi413288-bib-0006] Benjamini, Y. & Hochberg, Y. (1995) Controlling the false discovery rate: a practical and powerful approach to multiple testing. Journal of the Royal Statistical Society: Series B: Methodological, 57(1), 289–300. Available from: 10.1111/j.2517-6161.1995.tb02031.x

[emi413288-bib-0007] Bolyen, E. , Rideout, J.R. , Dillon, M.R. , Bokulich, N.A. , Abnet, C.C. , Al‐Ghalith, G.A. et al. (2019) Reproducible, interactive, scalable and extensible microbiome data science using QIIME 2. Nature Biotechnology, 37(8), 852–857. Available from: 10.1038/s41587-019-0209-9 PMC7015180 31341288

[emi413288-bib-0008] Bray, J.R. & Curtis, J.T. (1957) An ordination of the upland forest communities of southern Wisconsin. Ecological Monographs, 27(4), 326–349. Available from: 10.2307/1942268

[emi413288-bib-0009] Chen, F.‐L. , Wang, G.‐C. , Teng, S.‐O. , Ou, T.‐Y. , Yu, F.‐L. & Lee, W.‐S. (2013) Clinical and epidemiological features of *Chryseobacterium indologenes* infections: analysis of 215 cases. Journal of Microbiology, Immunology and Infection, 46(6), 425–432. Available from: 10.1016/j.jmii.2012.08.007 23022462

[emi413288-bib-0010] Chen, Q.‐L. , Cui, H.‐L. , Su, J.‐Q. , Penuelas, J. & Zhu, Y.‐G. (2019) Antibiotic resistomes in plant microbiomes. Trends in Plant Science, 24(6), 530–541. Available from: 10.1016/j.tplants.2019.02.010 30890301

[emi413288-bib-0011] Davidson, R.M. , Nick, S.E. , Kammlade, S.M. , Vasireddy, S. , Weakly, N. , Hasan, N.A. et al. (2022) Genomic analysis of a hospital‐associated outbreak of *Mycobacterium abscessus*: implications on transmission. Journal of Clinical Microbiology, 60(1), e01547‐21. Available from: 10.1128/JCM.01547-21 34705540 PMC8769749

[emi413288-bib-0012] Deekshit, V.K. & Srikumar, S. (2022) ‘To be, or not to be’—the dilemma of ‘silent’ antimicrobial resistance genes in bacteria. Journal of Applied Microbiology, 133, 2902–2914. Available from: 10.1111/jam.15738 35882476

[emi413288-bib-0013] Dijkshoorn, L. , Nemec, A. & Seifert, H. (2007) An increasing threat in hospitals: multidrug‐resistant *Acinetobacter baumannii* . Nature Reviews Microbiology, 5(12), 939–951. Available from: 10.1038/nrmicro1789 18007677

[emi413288-bib-0014] Faoro, H. , Oliveira, W.K. , Weiss, V.A. , Tadra‐Sfeir, M.Z. , Cardoso, R.L. , Balsanelli, E. et al. (2019) Genome comparison between clinical and environmental strains of *Herbaspirillum seropedicae* reveals a potential new emerging bacterium adapted to human hosts. BMC Genomics, 20, 630. Available from: 10.1186/s12864-019-5982-9 31375067 PMC6679464

[emi413288-bib-0015] Feldgarden, M. , Brover, V. , Haft, D.H. , Prasad, A.B. , Slotta, D.J. , Tolstoy, I. et al. (2019) Validating the AMRFinder tool and resistance gene database by using antimicrobial resistance genotype‐phenotype correlations in a collection of isolates. Antimicrobial Agents and Chemotherapy, 63(11), e00483‐19. Available from: 10.1128/AAC.00483-19 31427293 PMC6811410

[emi413288-bib-0016] Forsberg, K.J. , Reyes, A. , Wang, B. , Selleck, E.M. , Sommer, M.O. & Dantas, G. (2012) The shared antibiotic resistome of soil bacteria and human pathogens. Science, 337(6098), 1107–1111. Available from: 10.1126/science.122076 22936781 PMC4070369

[emi413288-bib-0017] Garcia‐Armisen, T. , Vercammen, K. , Passerat, J. , Triest, D. , Servais, P. & Cornelis, P. (2011) Antimicrobial resistance of heterotrophic bacteria in sewage‐contaminated rivers. Water Research, 45(2), 788–796. Available from: 10.1016/j.watres.2010.09.003 20870262

[emi413288-bib-0018] Gillings, M.R. , Gaze, W.H. , Pruden, A. , Smalla, K. , Tiedje, J.M. & Zhu, Y.‐G. (2015) Using the class 1 integron‐integrase gene as a proxy for anthropogenic pollution. Multidisciplinary Journal of Microbial Ecology, 9(6), 1269–1279. Available from: 10.1038/ismej.2014.226 PMC4438328 25500508

[emi413288-bib-0019] Gupta, S.K. , Padmanabhan, B.R. , Diene, S.M. , Lopez‐Rojas, R. , Kempf, M. , Landraud, L. et al. (2014) ARG‐ANNOT, a new bioinformatic tool to discover antibiotic resistance genes in bacterial genomes. Antimicrobial Agents and Chemotherapy, 58(1), 212–220. Available from: 10.1128/AAC.01310-13 24145532 PMC3910750

[emi413288-bib-0020] Hsueh, P. , Teng, L. , Yang, P. , Ho, S. , Hsieh, W. & Luh, K. (1997) Increasing incidence of nosocomial *Chryseobacterium indologenes* infections in Taiwan. European Journal of Clinical Microbiology and Infectious Diseases, 16(8), 568–574. Available from: 10.1007/BF02447918 9323467

[emi413288-bib-0021] Jain, C. , Rodriguez‐R, L.M. , Phillippy, A.M. , Konstantinidis, K.T. & Aluru, S. (2018) High throughput ANI analysis of 90K prokaryotic genomes reveals clear species boundaries. Nature Communications, 9, 5114. Available from: 10.1038/s41467-018-07641-9 PMC6269478 30504855

[emi413288-bib-0022] Jiang, X. , Ellabaan, M.M.H. , Charusanti, P. , Munck, C. , Blin, K. , Tong, Y. et al. (2017) Dissemination of antibiotic resistance genes from antibiotic producers to pathogens. Nature Communications, 8, 15784. Available from: 10.1038/ncomms15784 PMC5467266 28589945

[emi413288-bib-0023] Ju, F. , Beck, K. , Yin, X. , Maccagnan, A. , McArdell, C.S. , Singer, H.P. et al. (2019) Wastewater treatment plant resistomes are shaped by bacterial composition, genetic exchange, and upregulated expression in the effluent microbiomes. Multidisciplinary Journal of Microbial Ecology, 13(2), 346–360. Available from: 10.1038/s41396-018-0277-8 PMC6331547 30250051

[emi413288-bib-0024] Khare, D. , Kumar, R. & Acharya, C. (2020) Genomic and functional insights into the adaptation and survival of *Chryseobacterium* sp. strain PMSZPI in uranium enriched environment. Ecotoxicology and Environmental Safety, 191, 110217. Available from: 10.1016/j.ecoenv.2020.110217 32001422

[emi413288-bib-0025] Kirby, J.T. , Sader, H.S. , Walsh, T.R. & Jones, R.N. (2004) Antimicrobial susceptibility and epidemiology of a worldwide collection of *Chryseobacterium* spp.: report from the SENTRY Antimicrobial Surveillance Program (1997‐2001). Journal of Clinical Microbiology, 42(1), 445–448. Available from: 10.1128/JCM.42.1.445-448.2004 14715802 PMC321713

[emi413288-bib-0026] Lechner, M. , Findeiß, S. , Steiner, L. , Marz, M. , Stadler, P.F. & Prohaska, S.J. (2011) Proteinortho: detection of (co‐) orthologs in large‐scale analysis. BMC Bioinformatics, 12, 124. Available from: 10.1186/1471-2105-12-124 21526987 PMC3114741

[emi413288-bib-0027] Lerminiaux, N.A. & Cameron, A.D. (2019) Horizontal transfer of antibiotic resistance genes in clinical environments. Canadian Journal of Microbiology, 65(1), 34–44. 30248271 10.1139/cjm-2018-0275

[emi413288-bib-0028] Letunic, I. & Bork, P. (2021) Interactive Tree Of Life (iTOL) v5: an online tool for phylogenetic tree display and annotation. Nucleic Acids Research, 49(W1), W293–W296. Available from: 10.1093/nar/gkab301 33885785 PMC8265157

[emi413288-bib-0029] Levy, A. , Salas Gonzalez, I. , Mittelviefhaus, M. , Clingenpeel, S. , Herrera Paredes, S. , Miao, J. et al. (2018) Genomic features of bacterial adaptation to plants. Nature Genetics, 50(1), 138–150. Available from: 10.1038/s41588-017-0012-9 PMC5957079 29255260

[emi413288-bib-0030] Li, L.‐G. , Xia, Y. & Zhang, T. (2017) Co‐occurrence of antibiotic and metal resistance genes revealed in complete genome collection. Multidisciplinary Journal of Microbial Ecology, 11(3), 651–662. Available from: 10.1038/ismej.2016.155 PMC5322307 27959344

[emi413288-bib-0031] Lin, J.‐N. , Lai, C.‐H. , Yang, C.‐H. & Huang, Y.‐H. (2019) Differences in clinical manifestations, antimicrobial susceptibility patterns, and mutations of fluoroquinolone target genes between *Chryseobacterium gleum* and *Chryseobacterium indologenes* . Antimicrobial Agents and Chemotherapy, 63(5), e02256‐18. Available from: 10.1128/AAC.02256-18 30782983 PMC6496096

[emi413288-bib-0032] Lopeman, R.C. , Harrison, J. , Desai, M. & Cox, J.A. (2019) *Mycobacterium abscessus*: environmental bacterium turned clinical nightmare. Microorganisms, 7(3), 90. Available from: 10.3390/microorganisms7030090 30909391 PMC6463083

[emi413288-bib-0033] Mançano, S.M.C.N. , Campana, E.H. , Felix, T.P. , Barrueto, L.R.L. , Pereira, P.S. & Picão, R.C. (2020) Frequency and diversity of *Stenotrophomonas* spp. carrying *bla* _KPC_ in recreational coastal waters. Water Research, 185, 116210. Available from: 10.1016/j.watres.2020.116210 32731079

[emi413288-bib-0034] Murray, C.J. , Ikuta, K.S. , Sharara, F. , Swetschinski, L. , Aguilar, G.R. , Gray, A. et al. (2022) Global burden of bacterial antimicrobial resistance in 2019: a systematic analysis. Lancet, 399(10325), 629–655. Available from: 10.1016/S0140-6736(21)02724-0 35065702 PMC8841637

[emi413288-bib-0035] Mwanza, E.P. , Hugo, A. , Charimba, G. & Hugo, C.J. (2022) Pathogenic potential and control of *Chryseobacterium* species from clinical, fish, food and environmental sources. Microorganisms, 10(5), 895. Available from: 10.3390/microorganisms10050895 35630340 PMC9144366

[emi413288-bib-0036] O'Neill, J. (2016) Tackling drug‐resistant infections globally: final report and recommendations. UK: Government of the United Kingdom.

[emi413288-bib-0037] Parks, D.H. , Imelfort, M. , Skennerton, C.T. , Hugenholtz, P. & Tyson, G.W. (2015) CheckM: assessing the quality of microbial genomes recovered from isolates, single cells, and metagenomes. Genome Research, 25(7), 1043–1055. Available from: 10.1101/gr.186072.114 25977477 PMC4484387

[emi413288-bib-0038] Partridge, S.R. , Kwong, S.M. , Firth, N. & Jensen, S.O. (2018) Mobile genetic elements associated with antimicrobial resistance. Clinical Microbiology Reviews, 31(4), e00088‐17. Available from: 10.1128/CMR.00088-17 30068738 PMC6148190

[emi413288-bib-0039] Patel, J.B. , Cockerill, F. & Bradford, P.A. (2015) Performance standards for antimicrobial susceptibility testing: twenty‐fifth informational supplement. Malvern, PA: Clinical and Laboratory Standards Institute.

[emi413288-bib-0040] R Core Team . (2013) R: a language and environment for statistical computing. Vienna, Austria: R Foundation for Statistical Computing.

[emi413288-bib-0041] Rossi, E. , La Rosa, R. , Bartell, J.A. , Marvig, R.L. , Haagensen, J.A. , Sommer, L.M. et al. (2021) *Pseudomonas aeruginosa* adaptation and evolution in patients with cystic fibrosis. Nature Reviews Microbiology, 19(5), 331–342. Available from: 10.1038/s41579-020-00477-5 33214718

[emi413288-bib-0042] Sanz‐García, F. , Gil‐Gil, T. , Laborda, P. , Ochoa‐Sánchez, L.E. , Martínez, J.L. & Hernando‐Amado, S. (2021) Coming from the wild: multidrug resistant opportunistic pathogens presenting a primary, not human‐linked, environmental habitat. International Journal of Molecular Sciences, 22(15), 8080. Available from: 10.3390/ijms22158080 34360847 PMC8347278

[emi413288-bib-0043] Segata, N. , Börnigen, D. , Morgan, X.C. & Huttenhower, C. (2013) PhyloPhlAn is a new method for improved phylogenetic and taxonomic placement of microbes. Nature Communications, 4, 2304. Available from: 10.1038/ncomms3304 PMC3760377 23942190

[emi413288-bib-0044] Sereika, M. , Kirkegaard, R.H. , Karst, S.M. , Michaelsen, T.Y. , Sørensen, E.A. , Wollenberg, R.D. et al. (2022) Oxford Nanopore R10.4 long‐read sequencing enables the generation of near‐finished bacterial genomes from pure cultures and metagenomes without short‐read or reference polishing. Nature Methods, 19(7), 823–826. Available from: 10.1038/s41592-022-01539-7 35789207 PMC9262707

[emi413288-bib-0045] Sharma, P. , Gupta, S.K. , Diene, S.M. & Rolain, J.‐M. (2015) Whole‐genome sequence of *Chryseobacterium oranimense*, a colistin‐resistant bacterium isolated from a cystic fibrosis patient in France. Antimicrobial Agents and Chemotherapy, 59(3), 1696–1706. Available from: 10.1128/AAC.02417-14 25583710 PMC4325762

[emi413288-bib-0046] Srinivasan, V.B. , Rajamohan, G. & Gebreyes, W.A. (2009) Role of AbeS, a novel efflux pump of the SMR family of transporters, in resistance to antimicrobial agents in *Acinetobacter baumannii* . Antimicrobial Agents and Chemotherapy, 53(12), 5312–5316. Available from: 10.1128/AAC.00748-09 19770280 PMC2786332

[emi413288-bib-0047] Stasiak, M. , Maćkiw, E. , Kowalska, J. , Kucharek, K. & Postupolski, J. (2021) Silent genes: antimicrobial resistance and antibiotic production. Polish Journal of Microbiology, 70(4), 421–429. Available from: 10.33073/pjm-2021-040 35003274 PMC8702603

[emi413288-bib-0048] Tikhonova, E.B. , Yamada, Y. & Zgurskaya, H.I. (2011) Sequential mechanism of assembly of multidrug efflux pump AcrAB‐TolC. Chemistry & Biology, 18(4), 454–463. Available from: 10.1016/j.chembiol.2011.02.011 21513882 PMC3082741

[emi413288-bib-0049] Vandamme, P. , Bernardet, J.‐F. , Segers, P. , Kersters, K. & Holmes, B. (1994) New perspectives in the classification of the Flavobacteria: description of *Chryseobacterium* gen. nov., *Bergeyella* gen. nov., and *Empedobacter* nom. rev. International Journal of Systematic and Evolutionary Microbiology, 44(4), 827–831. Available from: 10.1099/00207713-44-4-827

[emi413288-bib-0050] Victor, M.P. , Das, L. & Das, S.K. (2022) The genome wide analysis deciphered virulence factors and secondary metabolites of *Chryseobacterium* and description of *Chryseobacterium indicum* sp. nov. bioRxiv. Available from: 10.1101/2022.03.19.484977

[emi413288-bib-0051] Vouga, M. & Greub, G. (2016) Emerging bacterial pathogens: the past and beyond. Clinical Microbiology and Infection, 22(1), 12–21. Available from: 10.1016/j.cmi.2015.10.010 26493844 PMC7128729

[emi413288-bib-0052] Wexler, H.M. (2007) Bacteroides: the good, the bad, and the nitty‐gritty. Clinical Microbiology Reviews, 20(4), 593–621. Available from: 10.1128/CMR.00008-07 17934076 PMC2176045

[emi413288-bib-0053] Xia, Y. , Sun, J. & Chen, D.‐G. (2018) Statistical analysis of microbiome data with R. Singapore: Springer.

[emi413288-bib-0054] Zhang, Q.‐Q. , Ying, G.‐G. , Pan, C.‐G. , Liu, Y.‐S. & Zhao, J.‐L. (2015) Comprehensive evaluation of antibiotics emission and fate in the river basins of China: source analysis, multimedia modeling, and linkage to bacterial resistance. Environmental Science and Technology, 49(11), 6772–6782. Available from: 10.1021/acs.est.5b00729 25961663

[emi413288-bib-0055] Zhang, R. , Dong, N. , Shen, Z. , Zeng, Y. , Lu, J. , Liu, C. et al. (2020) Epidemiological and phylogenetic analysis reveals *Flavobacteriaceae* as potential ancestral source of tigecycline resistance gene *tet*(X). Nature Communications, 11, 4648. Available from: 10.1038/s41467-020-18475-9 PMC7494873 32938927

