# Supplementary material for: Comparative resistomics analysis of multidrug‐resistant Chryseobacteria
Source: Environ Microbiol Rep. 2024 Jun 23;16(3):e13288. doi: 10.1111/1758-2229.13288 (PMC11194056; doi:10.1111/1758-2229.13288)
Supplement: Supplementary file 3 — Data S3. Supporting Information. [file EMI4-16-e13288-s002.docx]

**Supporting Information**

**for**

**Comparative** **Resistomics Analysis of Multidrug-resistant *Chryseobacteria***

Dung Ngoc Pham and Mengyan Li^*^

Department of Chemistry and Environmental Science, New Jersey Institute of Technology, Newark, New Jersey 07102, United States

*Address correspondence to Dr. Mengyan Li ([mengyan.li@njit.edu](mailto:mengyan.li@njit.edu))

Phone: +1-973-642-7095

Fax: +1-973-596-3586

**S1. RESULTS AND DISCUSSION**

**Majority of virulence factors independent to the origins**

The attachment of *Chryseobacteria* to host cells is associated with adherence genes, such as *Hsp60*, *EF-Tu*, and *IlpA*. It is important to note that *Hsp60* was found in all 215 *Chryseobacterium* genomes. *Hsp60* protein has been considered as a key virulent factor that stimulates adhesion in certain bacterial species such as *Mycobacterium tuberculosis*, *Clostridium difficile,* and *Helicobacter pylori*.^1^ The *EF-Tu* genes in *Mycobacterium pneumoniae* and *Pseudomonas aeruginosa* were responsible for binding fibronectin and plasminogen onto mammalian cells, respectively.^1^ The *IlpA* protein can induce cytokine production in human immune cells and act as an adhesion molecule as observed in *Vibrio vulnificus*.^2^

Following the primary surface adhesion, the growth of attached bacteria can be mediated by factors relevant to immune response, mobility (e.g., *flmH*), nutritional/metabolic traits (e.g., *mgtB*, *panD*). In *Chryseobacteria*, an immune response is predominant by the expression of capsule and formation of lipopolysaccharide (LPS) and lipooligosaccharide (LOS). Several highly abundant genes involved in the capsule biosynthesis pathway include *cap8E*, *cap8G,* and *tviB*. The expression of the type-8 capsular gene cluster (*cap8*) can promote the secretion of capsular polysaccharides, which was demonstrated effective to assist the pathogenic *Staphylococcus aureus* to avoid the phagocytic inactivation by the complement system and antibody generated from hosts.^1^ Using TEM, a recent study revealed the formation of capsular materials in the clinical isolate *Chryseobacterium gallinarum* strain MGC42.^3^ In addition, *acpXL*, *galE*, *rffG*, *wbpD*, *hisF2*, *lpxA*, *lpxD*, *rfbC*, *wbtL*, and *wbtF,* which are involved in the biosynthesis of LPS/LOS, were frequently detected among *Chrseobacterium* species. The formation of LPS/LOS often creates steric hindrance that is causal to the resistance of bacteria to antimicrobial effectors produced by the immune system, such as the LPS O-chain in pathogenic *Brucella*.^4^

Several virulence factors were associated with stress survival (*ClpP*, *UreB*, *UreG*, *KatG*, *KatAB*, *KatA*, *icl*), enabling bacterial pathogens to persist in host cells. *ClpP* was detected in all 215 *Chryseobacterium* isolates. It encodes proteases that can hydrolyse the peptide bond in the polypeptide chain, allowing pathogenic bacteria to uptake nutrients for survival in hosts.^5^ Expression of *ClpP*-coding proteases has been evident in clinical isolates *C. gleum* and *C. indologenes*.^6^ *UreA*, *UreB* and *UreG* were identified as key virulence factors for the persistence of pathogens in human stomach since they are involved in the hydrolysis of urea to ammonium and carbon dioxide.^7^ The presence of these virulent factors in *Chryseobacteria* may enhance their survival chance in the bladder system and result in cystitis, a common infectious disease frequently caused by pathogenic *C. indologenes*. *KatA*, *KatAB*, and *KatG* encode peroxidases that contribute to the inactivation of hydrogen peroxide, one of the host-derived reactive oxygen species that attack foreign cells and molecules. This function allows pathogenic bacteria to survive and proliferate in host cells.^8^ Isocitrate lyase encoded by *Icl* is an enzyme of the glyoxylate shunt enabling bacteria to utilize fatty acids when the availability of primary sources is limited.^9^ Further studies are needed to verify the roles and functions of these common virulent factors identified in *Chryseobacteria*.


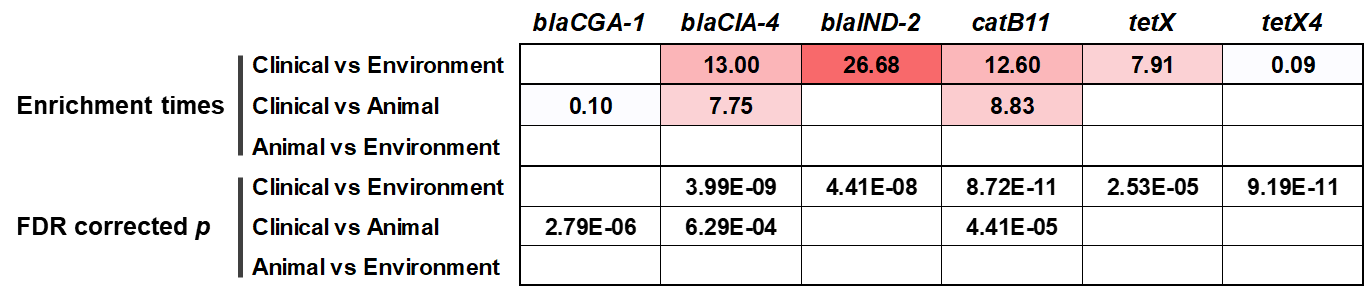


**Figure S1.** Significant differences in key ARGs between different sources with Fisher’s exact test *p* values.


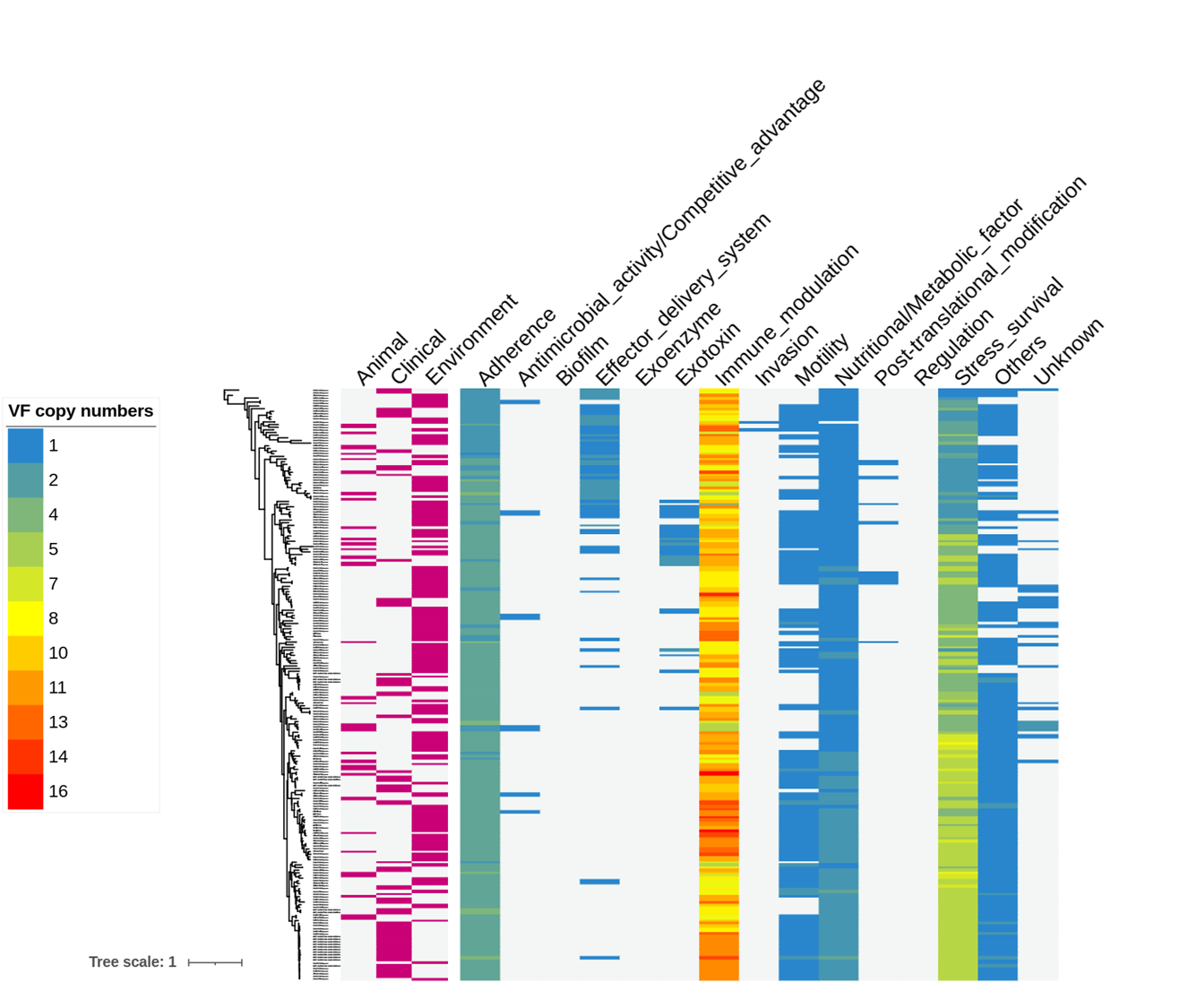


**Figure S2**. Phylogenetic tree of 215 *Chryseobacterium* genomes based on the virulence factor profile. The first column depicts the source of *Chryseobacterium* isolates, and the heat map shows the virulent factor abundance based on the categories on the top.

**Table S1.** A summary of quality of pre- and post-polishing genomes of 4 *Chryseobacterium* strains isolated in this study.

|  | ***Chryseobacterium* strain** | **Completeness (%)** | **Contamination (%)** |
| --- | --- | --- | --- |
| **Pre-polishing** | *C. indologenes* P3 | 70.10 | 2.51 |
|  | *C. indologenes* R7 | 71.59 | 2.43 |
|  | *C. bernardetii* L8 | 70.61 | 1.80 |
|  | *C. gleum* N14 | 74.28 | 0.49 |
| **Post-polishing** | *C. indologenes* P3 | 99.39 | 0.74 |
|  | *C. indologenes* R7 | 99.51 | 0.00 |
|  | *C. bernardetii* L8 | 98.53 | 0.25 |
|  | *C. gleum* N14 | 99.02 | 0.00 |

**Table S2.** Summary of *Chryseobacterium* strains carrying the *catB11-tetX* cluster*.*

| ***Chryseobacterium* strains** | **Origin** | **Genome ID (JGI)** | **Contig ID (JGI)** | **Genome/Contig ID (NCBI)** |
| --- | --- | --- | --- | --- |
| *Chryseobacterium taklimakanense* NCTC 13490 | Environment | 2765235860 | Ga0226281_11 | NZ_LT906465.1 |
| *Chryseobacterium nematophagum* JUb129 | Environment | 2881469986 | Ga0439802_01 | NZ_QWIU01000002.1 |
| *Chryseobacterium* sp. SNU WT5 | Animal | 2888282617 | Ga0443270_01 | NZ_CP041687.1 |
| *Chryseobacterium* sp. C3 | Clinical | NA | NA | NZ_JACLCU010000008.1 |
| *Chryseobacterium* sp. C2 | Clinical | NA | NA | NZ_JACLCT010000068.1 |
| *Chryseobacterium indologenes* Cl33 | Clinical | NA | NA | NZ_JACLDY010000004.1 |
| *Chryseobacterium indologenes* Cl21 | Clinical | NA | NA | NZ_JACLDL010000004.1 |
| *Chryseobacterium lactis* CL1 | Clinical | NA | NA | NZ_JACLEN010000066.1 |
| *Chryseobacterium lactis* CL2 | Clinical | NA | NA | NZ_JACLEO010000042.1 |

NA: not available

**Table S3.** Summary of *Chryseobacterium* strains carrying *abeS* and *RayT* orthologs.

| ***Chryseobacterium* strain** | **Origin** | **Genome ID (JGI)** | **Genome ID (NCBI)** | **Distance between *abeS* and *RayT* (kbp)** |
| --- | --- | --- | --- | --- |
| *Chryseobacterium* sp. CF365 | Populus root rhizosphere  (Environment) | 2582581278 |  | 4.6 |
| *Chryseobacterium* sp. Leaf394 | Arabidopsis leaf  (Environment) | 2643221937 |  | NA  >10 kbp**^a^** |
| *Chryseobacterium glaciei* IHBB 10212 | Glacier  (Environment) | 2721755524 |  | 1891.6 |
| *Chryseobacterium rhizoplanae* DSM 29371 | Maize rhizosphere (Environment) | 2724679795 |  | NA  >10 kpb**^a^** |
| *Chryseobacterium indoltheticum* G0211 | Soil  (Environment) | 2859301765 |  | 1712.8 |
| *Chryseobacterium indologenes* P3 | Activated sludge  (Environment) | 2886347146 |  | 4.0 |
| *Chryseobacterium gleum* F93, ATCC 35910 | Clinical | 2562617092 |  | 4.7 |
| *Chryseobacterium indologenes* mars15 | Clinical | 2744054951 |  | NA |
| *Chryseobacterium indologenes* FDAARGOS_337 | Clinical | 2816332311 |  | 4.0 |
| *Chryseobacterium indologenes* FDAARGOS_379 | Clinical | 2816332345 |  | 3.7 |
| *Chryseobacterium indologenes* FDAARGOS_510 | Clinical | 2847606532 |  | 4.7 |
| *Chryseobacterium indologenes* FDAARGOS_537 | Clinical | 2847611096 |  | 4.7 |
| *Chryseobacterium* sp. G0186 | Clinical | 2851044757 |  | 2160.9 |
| *Chryseobacterium gleum* 110146 | Clinical | 2851057275 |  | 3.9 |
| *Chryseobacterium indologenes* H5559 | Clinical | 2859305800 |  | 4.0 |
| *Chryseobacterium indologenes* | Clinical |  | GCF_014218815.1_ASM1421881v1 | 4.7 |
| *Chryseobacterium indologenes* | Clinical |  | GCF_014220145.1_ASM1422014v1 | 4.7 |
| *Chryseobacterium indologenes* | Clinical |  | GCF_014220155.1_ASM1422015v1 | 4.7 |
| *Chryseobacterium indologenes* | Clinical |  | GCF_014220165.1_ASM1422016v1 | 4.7 |
| *Chryseobacterium indologenes* | Clinical |  | GCF_014220395.1_ASM1422039v1 | 4.7 |
| *Chryseobacterium indologenes* | Clinical |  | GCF_014220445.1_ASM1422044v1 | 4.7 |
| *Chryseobacterium indologenes* | Clinical |  | GCF_014220465.1_ASM1422046v1 | 4.7 |
| *Chryseobacterium indologenes* | Clinical |  | GCF_014220695.1_ASM1422069v1 | 4.4 |

NA: Not available since *aadS* and *RayT* are located in two different contigs.

**a** Distance of *aadS* or *RayT* to the end of the contig is greater than 10 kbp

**Table S4**. A summary of virulent factors that were significantly different between clinical and environmental *Chryseobacterium* isolates.

| **Category** | **Gene name** | **Virulence factor ID** | **Total gene** | **Percentage of total virulence factors (%)** | **Odd number** | **Adjust p value** |
| --- | --- | --- | --- | --- | --- | --- |
| Immune modulation | *tviB* | VFG000431 | 29 | 0.6 | 8.26 | 1.77E-06 |
| Immune modulation | *tviB* | VFG037897 | 230 | 4.9 | 0.14 | 4.36E-06 |
| Others | *icl* | VFG001381 | 190 | 4.1 | 17.18 | 8.20E-05 |
| Stress survival | *KatG* | VFG001396 | 90 | 1.9 | 3.10 | 8.37E-04 |

**Reference**

1. Matos, A. L.; Curto, P.; Simões, I., Moonlighting in Rickettsiales: expanding virulence landscape. *Tropical medicine and infectious disease* **2022,** *7* (2), 32. PMID: 35202227, <https://doi.org/10.3390/tropicalmed7020032>.

2. Lee, K.-J.; Lee, N. Y.; Han, Y.-S.; Kim, J.; Lee, K.-H.; Park, S.-J., Functional characterization of the IlpA protein of Vibrio vulnificus as an adhesin and its role in bacterial pathogenesis. *Infection and immunity* **2010,** *78* (6), 2408-2417. PMID: 20308294, <https://doi.org/10.1128/IAI.01194-09>.

3. Gaur, M.; Dey, S.; Sahu, A.; Dixit, S.; Sarathbabu, S.; Zothanzama, J.; Sahoo, R. K.; Behera, D. U.; Subudhi, E., Characterization and Comparative Genomic Analysis of a Highly Colistin-Resistant Chryseobacterium gallinarum: a Rare, Uncommon Pathogen. *Frontiers in cellular and infection microbiology* **2022**, 924. PMID: 35909954, <https://doi.org/10.3389/fcimb.2022.933006>.

4. Haag, A. F.; Myka, K. K.; Arnold, M. F.; Caro-Hernández, P.; Ferguson, G. P., Importance of lipopolysaccharide and cyclic β-1, 2-glucans in Brucella-mammalian infections. *International journal of microbiology* **2010,** *2010*, PMID: 21151694, <https://doi.org/10.1155/2010/124509>.

5. Gaillot, O.; Pellegrini, E.; Bregenholt, S.; Nair, S.; Berche, P., The ClpP serine protease is essential for the intracellular parasitism and virulence of Listeria monocytogenes. *Molecular microbiology* **2000,** *35* (6), 1286-1294. PMID: 10760131, <https://doi.org/10.1046/j.1365-2958.2000.01773.x>.

6. Mwanza, E. P.; Hugo, A.; Charimba, G.; Hugo, C. J., Pathogenic potential and control of Chryseobacterium species from clinical, fish, food and environmental sources. *Microorganisms* **2022,** *10* (5), 895. PMID: 35630340, <https://doi.org/10.3390/microorganisms10050895>.

7. Montecucco, C.; Rappuoli, R., Living dangerously: how Helicobacter pylori survives in the human stomach. *Nature reviews molecular cell biology* **2001,** *2* (6), 457-466. PMID: 11389469, <https://doi.org/10.1038/35073084>.

8. Soler-Garcı́a, Á. A.; Jerse, A. E., A Neisseria gonorrhoeae catalase mutant is more sensitive to hydrogen peroxide and paraquat, an inducer of toxic oxygen radicals. *Microbial pathogenesis* **2004,** *37* (2), 55-63. PMID: 15312845, <https://doi.org/10.1016/j.micpath.2004.04.007>.

9. McKinney, J. D.; Zu Bentrup, K. H.; Muñoz-Elías, E. J.; Miczak, A.; Chen, B.; Chan, W.-T.; Swenson, D.; Sacchettini, J. C.; Jacobs, W. R.; Russell, D. G., Persistence of Mycobacterium tuberculosis in macrophages and mice requires the glyoxylate shunt enzyme isocitrate lyase. *Nature* **2000,** *406* (6797), 735-738. PMID: 10963599, <https://doi.org/10.1038/35021074>.
